# Determination of Oxidized Lipids in Commonly Consumed Foods and a Preliminary Analysis of Their Binding Affinity to PPARγ

**DOI:** 10.3390/foods10081702

**Published:** 2021-07-22

**Authors:** Joanna Skinner, Payal Arora, Nicole McMath, Meera Penumetcha

**Affiliations:** Department of Nutrition, Byrdine F. Lewis College of Nursing and Health Professions, Georgia State University, Atlanta, GA 30302, USA; jpskinner@comcast.net (J.S.); payal.arora@doh.nj.gov (P.A.); nicole.mcmath@valetliving.com (N.M.)

**Keywords:** food lipids, lipid oxidation, peroxisome proliferator receptor gamma, peroxide value, conjugated dienes, aldehydes

## Abstract

Foods rich in poly unsaturated fatty acids (PUFA) are vulnerable to oxidation. While it is well established that endogenously derived oxidized lipids are ligands of the transcription factor PPARγ, the binding ability of diet-derived oxidized lipids is unknown. Our two-fold objective was to determine the oxidized lipid content and PPARγ binding ability of commonly consumed foods. Extracted food lipids were assayed for the peroxide value, conjugated dienes, and aldehydes, and PPARγ binding was assessed by an in vitro PPARγ ligand screening assay. Oxidized lipids were present in all foods tested at the time of purchase, and oxidation did not increase during storage. The peroxide values for walnuts, sunflower seeds, and flax meal were significantly lower at the end of three months as compared to the day of purchase (peroxide value: 1.26 ± 0.13 vs. 2.32 ± 0.4; 1.65 ± 0.23 vs. 2.08 ± 0.09; 3.07 ± 0.22 vs. 9.94 ± 0.75 mEq/kg fat, *p* ≤ 0.05, respectively). Lipids extracted from French fries had the highest binding affinity (50.87 ± 11.76%) to PPARγ compared to other foods. Our work demonstrates that oxidized lipids are present in commonly consumed foods when purchased, and for the first time demonstrates that some contain ligands of PPARγ.

## 1. Introduction

Polyunsaturated fatty acids (PUFA) are vulnerable to oxidation. Foods that contain PUFAs undergo lipid oxidation during food preparation, processing, and storage. This non-enzymatic thermal oxidation of PUFA results in the formation of primary oxidation products, which are hydroperoxy and hydroxy fatty acids of the parent fatty acid. For example, oxidation of linoleic acid, the most common fatty acid consumed in theUnited States (US) results in the formation of 13-hydroperoxy linoleic acid (13-HPODE), which is then reduced to13-hydroxy linoleic acid (13-HODE). With subsequent increase in time and/or temperature, these primary products of oxidation undergo scission between carbon atoms and form short, secondary products of oxidation such as aldehydes, ketones, and epoxy compounds. While the impact of oxidation, both enzymatic and non-enzymatic, of endogenous lipids has been investigated extensively [1,2,3,4], studies investigating the risk of chronic diseases by dietary oxidized lipids (DOL) (in other words, exogenous lipids), are just beginning to emerge [5,6,7]. While the digestibility of DOL is lower than that of unoxidized lipids [8,9], they are absorbed and transported in the plasma [10] and undergo cellular metabolism. Studies, primarily in animal models using heated vegetable oils as a source of DOL have demonstrated that DOL consumption is associated with hypertension [11], atherosclerosis [12,13], and glucose intolerance [6,14]. To translate the work from animals to humans, it would be necessary to know the oxidized lipid content of commonly consumed foods.

We propose that Peroxisome Proliferator Activated Receptor Gamma (PPARγ), a transcription factor, could be a physiological link between DOL and adipose tissue homeostasis. This transcription factor is essential for adipocyte differentiation and maintenance of healthy adipose tissue [15,16] and is a mediator of inflammation [17]. Furthermore, pharmaceutical activation of PPARγ by thizolidienediones, agonists of PPARγ, promotes glucose tolerance. Of great interest is the demonstration that oxidized fatty acids as compared to unoxidized fatty acids are excellent ligands of PPARγ [18,19], suggesting a role for oxidized fatty acids in adipose tissue and glucose homeostasis. However, it is not known if food-derived oxidized lipids are ligands of PPARγ.

Thus, the first objective of this study was to determine the levels of oxidized lipids in commonly consumed foods during storage, similar to that in a household pantry, and second to examine the PPARγ ligand binding ability of lipids extracted from these foods in an in vitro assay. Because thermal oxidation of vegetable oil has already been studied, heated vegetable oil was used as the reference in this study.

## 2. Materials and Methods

### 2.1. Materials

Except for the vegetable oil, all foods analyzed for this project were purchased in Atlanta-area supermarkets and fast-food outlets. Soybean vegetable oil was an in-kind donation from Welch, Holme & Clark Co., Inc. (Newark, NJ, USA). The SafTest PeroxySafe and AldeSafe kits were obtained from MP Biomedicals (Solon, OH, USA). The PPARγ ligand screening assay kit was procured from Cayman Chemicals (Ann Arbor, MI, USA). All remaining chemicals were purchased from Sigma (St. Louis, MO, USA).

### 2.2. Thermal Oxidation of Vegetable Oil

The soybean oil was refined and contained 53.7% linoleic acid, 23.3% oleic acid, 10.5% palmitic acid, and 7.3% linolenic acid as per the manufacturer’s data sheet. Oxidation of the soybean oil was promoted by heating. Before the heating process, approximately 200 mL of unheated oil (UHO) was stored under nitrogen at −80 °C. The rest of the oil was heated to 195 °C, in a 1000 mL glass beaker on a heating stand, and the temperature was maintained for the duration of the heating process. Aliquots of 200 mL oil were removed from heating at 3, 6, and 9 h. After removal from the heat, each aliquot of oil was cooled at room temperature and then stored under nitrogen at −80 °C until analysis.

### 2.3. Room-Temperature Foods (RTF) and Fried Foods

Five types of packaged foods and supplements were purchased as close in time as possible and stored at room temperature (24 ± 2 °C). For the purpose of this study, these were considered “room-temperature foods” (RTF) and included raw walnuts, raw sunflower seeds, flax meal, omega 3 fish oil capsules, and DHA&ARA-enriched infant formula. All RTF were purchased in local grocery stores in the Atlanta area (Georgia, USA) and only one brand of each item was used for the study. Un-weighed samples of each of the RTF were placed into airtight conical tubes and stored under nitrogen at −80 °C to prevent further oxidation. The remaining RTF were left in their original packaging (to replicate conditions in a consumer’s home) for one month after purchase. At the end of that month, aliquots of each food were placed in the −80 °C freezer, with the remainder of the RTF left in the original packaging. This process was repeated at two and three months from the purchase date. In addition to the RTF, fried foods, including French fries and chicken nuggets, were purchased on a single day from the same fast-food establishment. After purchase, the fried foods were pulverized in a Braun coffee mill (Kronberg, Germany) and stored under nitrogen at −80 °C until lipid extraction the following day.

### 2.4. Extraction of Lipids

Prior to extraction, foods were thawed for a maximum of 10 min. Foods not already in ground or oil form were ground in a coffee mill, and 1.5 g aliquots of each food was extracted. With the exception of the chicken nuggets, oils were extracted from foods using a solvent containing hexane and isopropanol (HIP) in a 3:2 ratio according to the modified method of Hara and Radin [20]. For chicken nuggets, a chloroform and methanol (2:1) solvent was used in a modified Folch method [21]. In both methods, each 1.5 g of food sample was homogenized in a 20 mL solvent using a Polytron homogenizer (Kinematica AG, Lucerne, Switzerland) at 19,000 rpm for a total of 1.5 min. When all portions of a given sample were homogenized, the homogenate was combined and filtered through 90 mm filter paper (Whatman plc, Kent, UK) into a glass flask. The filtrate was transferred to a separatory funnel and 12 mL of 0.467 M Na_2_SO_4_ (anhydrous) was added for every gram of food. The separatory funnel was shaken to thoroughly mix the contents and left untouched for 10–20 min to allow separation of the polar and non-polar layers. The lipid layer was removed and poured into a 30 mL test tube, which was then placed in an Organomation nitrogen evaporator (Organomation, Berlin, MA, USA) water bath (30 °C) to evaporate the solvent. One hundred microliter aliquots of each of the extracted lipid samples were weighed and then stored under nitrogen at −80 °C.

### 2.5. Quantifying the Products of Oxidation

#### 2.5.1. Primary Oxidation Products

The peroxide value (PV) and conjugated dienes (CD) were used to indicate the presence of the primary products of oxidation. To determine CD, the sample oil was solubilized in hexane to create a 1% solution. This solution was diluted as necessary by a process of trial and error to achieve spectrophotometric readings in the target absorbance range of 0.2–0.8 at 234 nm. Hexane was used as the blank, and all samples were tested in triplicate. Oil samples were read in an Epoch spectrophotometer (BioTek, Winooski, VT, USA). CD levels were expressed as absorbance of a 1% solution at 234 nm E_234_^1%^. All oil samples were tested in triplicate, and the test was repeated twice. The concentration of lipid peroxides was determined using the SafTest PeroxySafe kit. Briefly, 200 μL of the calibrator, control or diluted sample was placed in a glass tube and then reagents were added as per manufacturer’s protocol. Each sample was vortexed at high speed for 10 s and incubated at room temperature for 10 min on a rocker followed by an immediate reading of the absorbance in an Epoch spectrophotometer at 570 nm and 690 nm. Each time the standards were assayed first and then each sample was diluted to assure that the absorbance values of the samples fell within the range of the calibration curve. All samples were run in triplicate and were repeated twice.

#### 2.5.2. Secondary Oxidation Products

For measuring the level of aldehydes in the samples, an AldeSafe kit was used. The concentration of Thiobarbituric Acid Reactive Substances (TBARS), which include aldehydes, was determined using the SafTest Aldesafe kit. Briefly, 400 μL of the calibrator, control or diluted sample was placed in a glass tube and then reagents, along with TBA, were added as per the manufacturer’s protocol. Each sample was vortexed at high speed for 10 s and incubated in a 40 °C water bath for 20 min followed by an immediate reading of the absorbance in an Epoch spectrophotometer at 550 nm and 690 nm. Each time the standards were assayed first and then each sample was diluted to assure that the absorbance values of the samples fell within the range of the calibration curve. All samples were run in triplicate and were repeated twice.

### 2.6. Determining the Binding Affinity of Extracted Lipids for PPARγ

To determine the binding affinity of the extracted lipid samples for PPARγ, a fluorescence polarization-based PPARγ ligand screening assay kit was used. In this assay a ligand of PPARγ is conjugated to a fluorescein probe, which is displaced in the presence of PPARγ agonist/ligand, thereby decreasing fluorescence (measured in RFU). Four concentrations of each sample were prepared in dimethyl sulfoxide (DMSO): 1%, 0.1%, 0.01%, and 0.001%. A 384-well plate was prepared according to the manufacturer’s protocol, and all assays were performed in triplicate and repeated at least twice. The plates were read at 450 nm in a Victor^3^ spectrophotometer (Perkin Elmer, Waltham, MA, USA). Serial dilutions of the standard (rosiglitazone) and a known PPARγ antagonist (GW9662) were read concurrently with the samples. Unheated and heated vegetable oil samples were hydrolyzed by alkaline hydrolysis (see Section 2.7) and prepared as serial dilutions, beginning with a 1% solution, and diluted by half with DMSO to yield a total of 12 concentrations.

### 2.7. Alkaline Hydrolysis of Vegetable Oil

After an initial assay failed to show the binding affinity of the vegetable oil for PPARγ, unheated and vegetable oils heated for 3, 6, and 9 h were subjected to KOH hydrolysis. The hydrolysis was conducted according to the method described by Woo and Kim [22]. Briefly, 0.8 mg of oil was solubilized in 200 µL of 1 M KOH solution in a glass tube and heated in a water bath at 75 °C for 1 h with the glass tubes tightly capped. After cooling at room temperature, the fatty acids were extracted by washing first with water and then with diethyl ether.

### 2.8. Data Analysis

All data were analyzed with SPSS version 27.0 (IBM Corporation, Armonk, NY, USA) and tested for normality. The Mann–Whitney U test was used to compare mean levels of oxidized lipids between the purchase date and 3-month storage. The Kruskal–Wallis test followed by the Mann–Whitney U test was used to compare mean differences in lipid oxidation products in vegetable oils at different timepoints. A *p*-value of 0.05 was considered to be statistically significant for all statistical tests with the exception of the multiple comparisons that were done to compare the PPARγ binding affinity (*p*-value = 0.05/6 = 0.008) between seven food items.

## 3. Results

### 3.1. Lipid Oxidation Products in Soy Vegetable Oil

**Figure 1:**
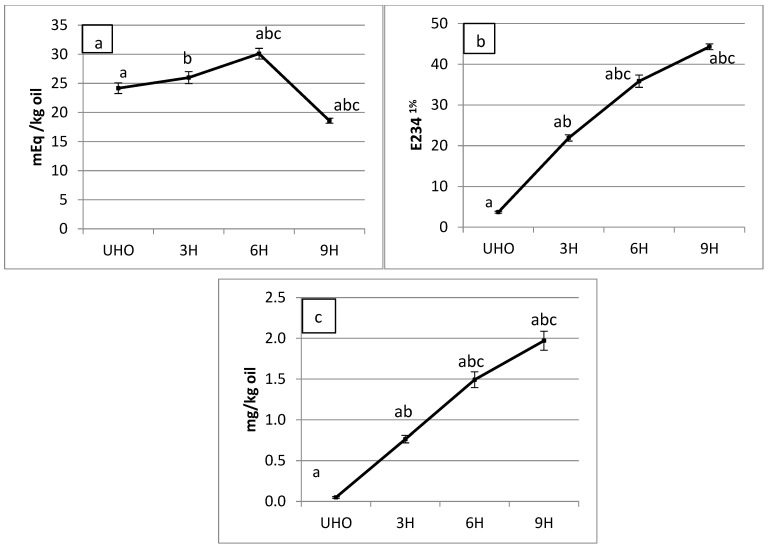
Products of lipid oxidation in unheated and heated vegetable oil. Mean ± SEM peroxide value (**a**), conjugated dienes (**b**) and aldehydes (**c**) of soybean oil unheated (UHO) or heated for 3H, 6H or 9H. Mean differences were determined by Kruskal Wallis test followed by pairwise comparisons with Mann–Whitney U. Means with the same letter differ significantly from each other (*p* ≤ 0.05).

Vegetable oil was used as a reference for lipid oxidation. As expected, heating of the oil caused an increase in lipid oxidation. Peroxide values (PV) increased until the 6 hour (6H) time point and then decreased (Figure 1a). There was an incremental accumulation of conjugated dienes (CD) and aldehydes with increased heating, and this increase was statistically significant between each of the time points (Figure 1b,c).

### 3.2. Lipid Oxidation Products in RTF and Fried Foods

**Figure 2:**
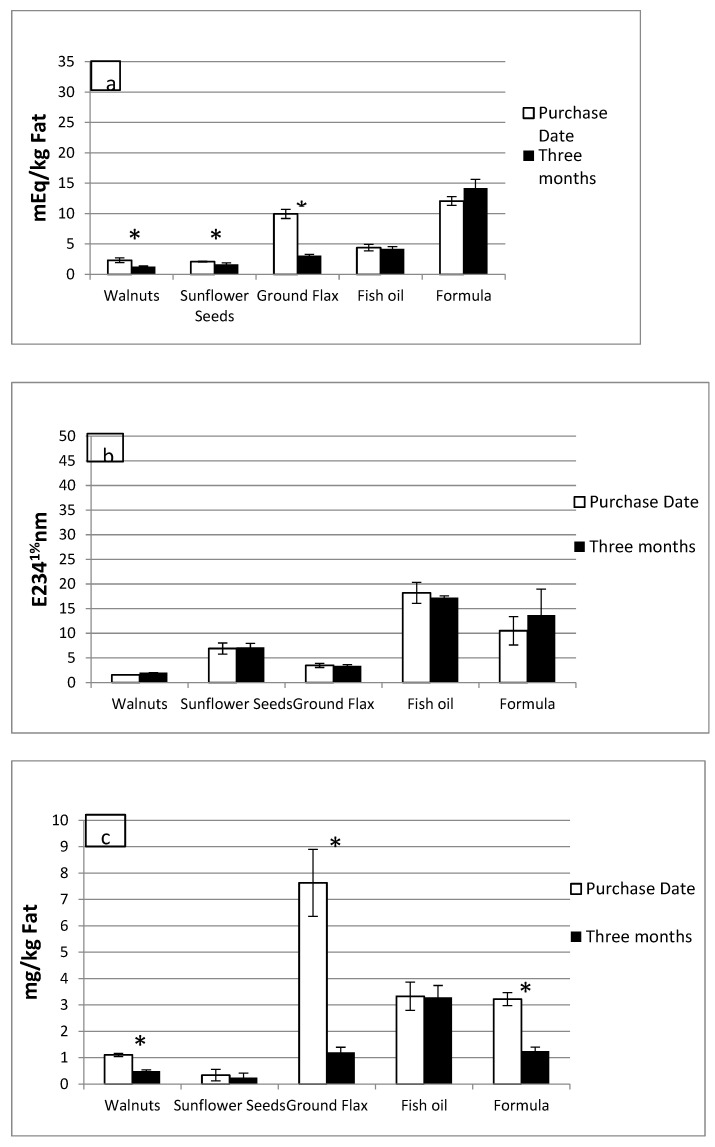
Products of lipid oxidation in room-temperature foods. Mean ± SEM of the peroxide value (**a**), conjugated dienes (**b**), and aldehydes (**c**) in room-temperature foods (RTF) on the purchase date (white bars) and after three months (black bars). Mean differences between the two time points were compared with the Mann–Whitney U test. * *p* ≤ 0.05.

Commonly consumed foods had lipid oxidation products at the time of purchase. As illustrated in Figure 2, mean PV, CD, and aldehydes did not increase significantly, after three-month storage, in any of the room-temperature foods (RTF) tested. Surprisingly, walnuts, sunflower seeds, and flax meal had significantly lesser amounts of peroxides (1.26 ± 0.13 vs. 2.32 ± 0.4; 1.65 ± 0.23 vs. 2.08 ± 0.09; 3.07 ± 0.22 vs. 9.94 ± 0.75 mEq/kg fat respectively; *p* ≤ 0.05) after three months of storage as compared to the purchase date (Figure 2a). There were no significant changes in the CD values (Figure 2b) of any of the RTF after three months of storage. The aldehyde content followed the same trend as the peroxide values for walnuts (Figure 2c), flax meal, and enriched infant formula with a significant decrease after three months of storage as compared to the purchase date (0.49 ± 0.05 vs. 1.1 ± 0.06; 1.2 ± 0.2 vs. 7.63 ± 1.27; 1.24 ± 0.16 vs. 3.22 ± 0.25 mg/kg fat respectively; *p* ≤ 0.05). Lipids extracted from French fries had a mean PV of 1.64 ± 0.29 mEq/kg fat; CD of 19.27 ± 0.36 E_234_^1%^, and aldehydes of 8.88 ± 0.15 mg/kg fat. Chicken nuggets had a mean PV of 2.05 ± 0.18 mEq/Kg fat, CD of 16.87 ± 0.18 E_234_^1%^, and aldehydes of 5.52 ± 0.14 mg/kg fat.

### 3.3. PPARγ Ligand Binding

**Figure 3:**
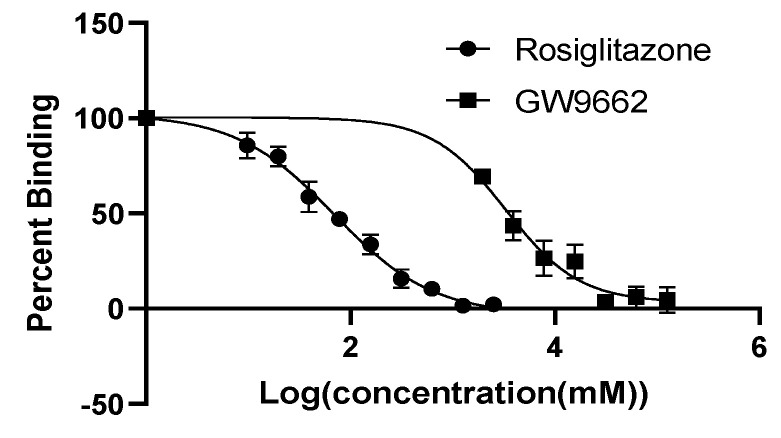
PPARγ ligand binding. PPARγ binding of rosiglitazone (circle) and GW9662 (square) was determined by an in-vitro ligand screening assay kit. The four-parameter logistic regression (4PL) model was used to fit the curves with GraphPad Prism 9.0(GraphPad Software, San Diego, CA, USA).

**Figure 4:**
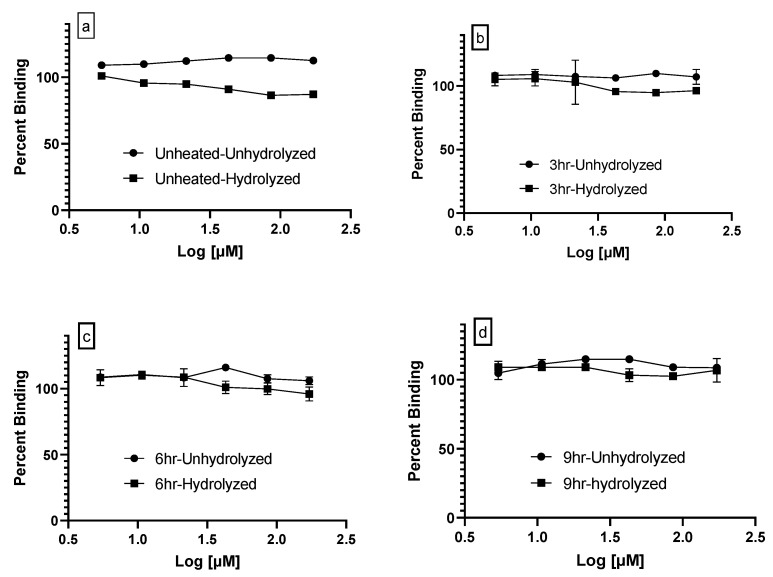
PPARγ binding of unhydrolyzed and hydrolyzed vegetable oil. Mean ± SEM of PPARγ ligand binding curves of unheated oil (**a**), 3H heated oil (**b**), 6H heated oil, (**c**) and 9H heated oil (**d**). Within each graph, the dark circle represents unhydrolyzed and the dark square represents oil subjected to alkali hydrolysis. The molar concentration of soy oil was determined in previously published work. There were no significant differences in the binding affinity between the unhyrolyzed and the hydrolyzed oils in the unheated and heated oils.

**Figure 5:**
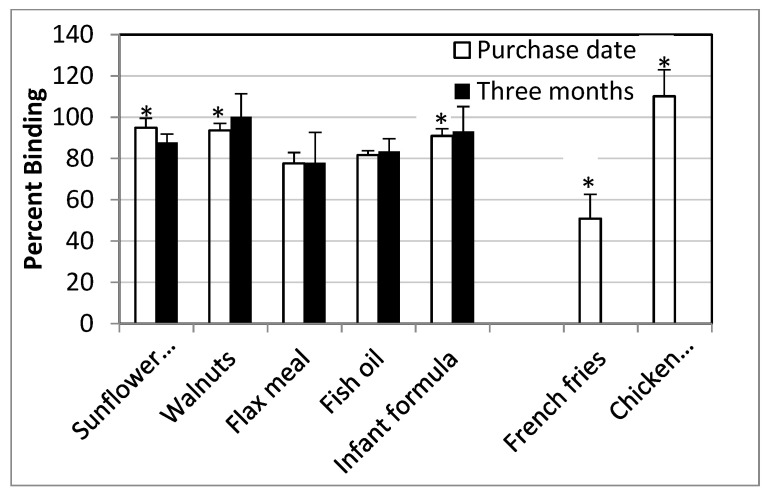
Percent PPARγ binding by lipids extracted from foods. Means ± SEM of the PPARγ binding of lipids extracted from various foods at a 1% concentration on the purchase date (white bars) and after 3-month storage (black bars) at room temperature were not significantly different. * Mean binding of French fries was significantly different than that of sunflower seeds, walnuts, infant formula and chicken nuggets.

The PPARγ ligand-binding assay is a competitive assay in which the replacement of a fluorescent probe by a ligand results in decreased fluorescence units but indicates increased binding affinity, which is expressed as binding between 0–100%. Rosliglitizone, a PPARγ agonist, demonstrated an IC_50_ of 0.069 mM while GW9662, an antagonist, had an IC_50_ of 3.3 mM (Figure 3). Both unheated and heated vegetable oils had a percent binding close to one hundred, indicating little to no affinity for PPARγ. Alkaline hydrolysis of unheated and heated oils improved the binding at the most by 15%, but this was not statistically significant (Figure 4). Lipids extracted from RTF demonstrated PPARγ binding in the range of 79–100% but did not differ much between the purchase date as compared to three months (Figure 5). French fries demonstrated the lowest PPARγ binding affinity at 50.87 ± 28.81% followed by flax meal at 77.62 ± 12.86% on the purchase date.

## 4. Discussion

This study demonstrates that commonly consumed foods such as walnuts, sunflower seeds, flax meal, fish oil capsules, DHA & ARA enriched infant formula, French fries, and chicken nuggets contain varying amounts of both primary and secondary products of lipid oxidation at the time of purchase. Furthermore, unlike the vegetable oil tested in this study, which demonstrated a continuous increase in lipid oxidation products with time and thermal stress (with the exception of lipid hydroperoxides), products of lipid oxidation did not increase significantly with time in walnuts, sunflower seeds, flax meal, and enriched infant formula. Instead, the peroxide and aldehyde values were significantly lower after three months of storage while the conjugated dienes did not change. It is possible that under the storage conditions used in this study, the decomposition of peroxides was greater than their generation. A similar explanation would support the decrease in the aldehyde content. In other words, aldehydes, due to their volatile nature, although present in foods at time of purchase, might evaporate over time. This suggests that the initiation of lipid oxidation is minimal under conditions that mimic storage in a food pantry.

The quantity of lipid oxidation products in the RTF were both similar and dissimilar to previous studies. The peroxide value of walnuts on the purchase day was higher by 43% and 330% compared to those reported by Aghdam and Chee [23,24], respectively. These higher levels could be attributed to the fact we used store-bought walnuts with an unknown harvest date while in both previous studies, walnuts were freshly harvested. The mean PV of the fish oil estimated in this study, 4.39 meq/kg, is similar to that purchased in Australia (range 2.77–4.63 meq/kg) [25] and the study by Zyoud and colleagues [26], who reported a combined mean PV of 6.4 meq/kg for fish oil capsules purchased in the USA, Canada, India, and the EU. The fact that our study demonstrated that the PV stayed the same after storage for three months and was within the maximum reference limit (5 meq/kg fat) set by the Global Organization of EPA and DHA (GOED) suggests that omega rich fish oil capsules are oxidatively stable in storage. Hussain and colleagues [27] reported no significant changes in the PV, conjugated dienes, and aldehyde content of flax meal stored at 20–25 °C for 90 days. This is different from the present study where we report a significant decrease in the PV and aldehyde values in flax meal after storage. In addition, we report a much higher amount of PV on the purchase date, 9.94 meq/kg as compared to 0.15 meq/kg, and this could be explained by the fact that flax meal in the previous study was freshly made from seeds while we purchased flax meal from the grocery store. Of the RTF that were tested, enriched infant formula had the highest amount of PV on the purchase date but did not change significantly after 3-month storage. Previous studies have also demonstrated that omega 3-enriched infant formulas contain higher amounts of lipid hydroperoxides and MDA as compared to un-enriched infant formula [28]. The levels of lipid oxidation products in French fries and chicken nuggets were similar to those in the RTF and not higher as we originally hypothesized. However, since we did not know the type of oil, its reuse, or frying temperature, it is difficult to examine the results within the context of work from other laboratories. Since these results suggest that oxidized lipids are already present in foods at the time of purchase, clinical trials should be conducted to study their metabolic fate in the short term and health effects in the long term.

It is well known that food processing promotes lipid oxidation but can be reduced in the presence of antioxidants, whether added or naturally occurring, in the food matrix [29]. For example, fish oil, which had the highest amount of vitamin E (2 IU/g PUFA), had the lowest levels of lipid oxidation products as compared to flax meal and enriched infant formula, which contained 1.46 IU and 0.01 IU of vitamin E/g PUFA, respectively. On the other hand, despite the presence of vitamin E and C, enriched infant formula had the highest PV per serving (0.1 mEq/serving) owing to the presence of the highest amount of the prooxidant iron (1.75 mg/g PUFA) as compared to all other foods. Infant formulas in the US are mandated to be enriched with iron, and the more recent trend of enriching formula with omega 3 PUFA for its purported brain health benefits needs to be revisited. This is especially relevant in light of a recent meta-analysis that demonstrated a lack of evidence of correlation between omega 3 fatty acid supplementation and infant health, especially in the areas of brain health and growth [30]. Nonetheless, addition of novel food-based antioxidants to PUFA-rich foods should become a common practice in the food industry.

It is well established that fatty acids and their derivatives are agonists of PPARγ, but more recent studies have demonstrated that nutrients other than fatty acids, such as polyphenols, arginine, and glutamine, are also ligands of this promiscuous receptor [18]. To our knowledge, we are the first to report on the ligand binding ability of lipids extracted from commonly consumed foods. Of the oils tested, oil extracted from French fries demonstrated decreased binding (increased affinity) to PPARγ, and this was significantly different from all other foods that were tested with the exception of flax meal and fish oil. Since only primary products of lipid oxidation such as 13-HODE, 9-HODE, and 13-oxoHODE are ligands of PPARγ, we hypothesized that foods with higher CD values (representing hydroxy, epoxy, and oxo-fatty acids) would have higher binding affinities. However, we were unable to see this connection as demonstrated by the fact even though French fries, chicken nuggets, and enriched fish oil had similar amounts of CD 

(15–20 E_234_^1^), only French fries demonstrated the lowest binding affinity. It is well known that when vegetable oils with a higher PUFA content are subjected to higher temperatures, as in frying, they yield more oxidized PUFAs [31,32]. This effect is demonstrated as a decrease in un-oxidized PUFA and MUFA contents of vegetable oils with increasing time and temperature [31]. Thus, it is plausible that the fries tested in this study were prepared with an oil with a higher PUFA content as compared to the oil used to prepare the chicken nuggets. The higher PUFA content might have generated a sufficient amount of oxidized lipids to demonstrate PPARγ binding. Furthermore, emerging studies suggest that nutrients, other than oxidized fatty acids, such as curcumin, capsaicin, and a variety of flavonoids, are agonists of PPARγ [33]. In other words, we cannot rule out that fat extracted from this one brand of French fries contained other PPARγ ligands. The lack of binding seen with most foods could be explained by the idea that oxidized intact triglycerides, polymers, and dimers are not ligands of PPARγ. However, it is known that lipid oxidation leads to some hydrolysis of fatty acids, thereby generating ligands for PPARγ. To test this further, we subjected unheated and heated vegetable oils to alkali hydrolysis but failed to see any improvement in the binding affinity. It is possible that the assay kit used in this study was sub-optimal for screening samples with low levels of PPARγ ligands. We reached this conclusion because as stated in the assay kit description, the IC_50_ for rosiglitazone was in the mM range, which is much higher than other assays that demonstrate IC_50_ values in the lower nM range for this PPARγ agonist.

One of the limitations of this study is the use of kits [34] to determine the lipid peroxide content in foods instead of the AOCS method. While this might have been less optimal in quantifying absolute amounts of lipid peroxides, it is still an accurate reflection of the relative amounts of lipid peroxides in foods determined in this study. Another possible limitation is that we did not determine the fatty acid composition of the extracted lipids. While it is true that standard methodology such as gas chromatography exists for quantifying fatty acids, methods to determine individual oxidized fatty acids, such as hydroxy, hydroperoxy, epoxy, and keto fatty acids from a complex mixture, are methodologically challenging and were not available during the study. We are glad to report that recently we were able to determine oxidized fatty acids in fat extracted from potatoes that were either oven-fried or deep-fried using three different vegetable oils by redox–lipidomics [32].

## 5. Conclusions

Common practices of food handling result in the oxidation of PUFA-rich foods. Based on the foods tested in this study, storage of foods at room temperature for three months does not increase lipid oxidation. However, the fact that products of lipid oxidation were present on the date of purchase suggests that the US population consumes oxidized lipids. Oxidized lipids extracted from foods in this study, for the most part, with the exception of French fries and flax meal, did not bind to PPARγ. This lack of binding remained despite our efforts to release free fatty acids from oxidized triglycerides. One possible explanation for this could be that the concentration of the oxidized lipids was too low to be detected by this kit. We feel that employing a combination of techniques such as thermal shift assay, ANS fluorescence quenching, and food lipid-bound crystallographic studies of the PPARγ ligand binding domain [35] might help overcome this problem. Subsequently, if future studies are able to establish that commonly consumed foods harbor oxidized lipids that act as PPARγ ligands, then future studies can investigate the cellular/molecular mechanisms by which these dietary oxidized lipids alter adipose tissue metabolism and glucose homeostasis.

## Data Availability

Not applicable.

## References

[1] Lee S., Birukov K.G., Romanoski C.E., Springstead J.R., Lusis A.J., Berliner J.A. (2012). Role of phospholipid oxidation products in atherosclerosis. Circ. Res..

[2] Zhong S., Li L., Shen X., Li Q., Xu W., Wang X., Tao Y., Yin H. (2019). An update on lipid oxidation and inflammation in cardiovascular diseases. Free Radic. Biol. Med..

[3] Florens N., Calzada C., Lyasko E., Juillard L., Soulage C.O. (2016). Modified Lipids and Lipoproteins in Chronic Kidney Disease: A New Class of Uremic Toxins. Toxins.

[4] Sottero B., Rossin D., Poli G., Biasi F. (2018). Lipid Oxidation Products in the Pathogenesis of Inflammation-related Gut Diseases. Curr. Med. Chem..

[5] Sayon-Orea C., Martinez-Gonzalez M.A., Gea A., Flores-Gomez E., Basterra-Gortari F.J., Bes-Rastrollo M. (2014). Consumption of fried foods and risk of metabolic syndrome: The SUN cohort study. Clin. Nutr..

[6] Sayon-Orea C., Carlos S., Martínez-Gonzalez M.A. (2015). Does cooking with vegetable oils increase the risk of chronic diseases?: A systematic review. Br. J. Nutr..

[7] Gadiraju T., Patel Y., Gaziano J., Djoussé L. (2015). Fried Food Consumption and Cardiovascular Health: A Review of Current Evidence. Nutrients.

[8] Olivero-David R., Paduano A., Fogliano V., Vitaglione P., Bastida S., González-Muñoz M.J., Benedí J., Sacchi R., Sánchez-Muniz F.J. (2011). Effect of Thermally Oxidized Oil and Fasting Status on the Short-Term Digestibility of Ketolinoleic Acids and Total Oxidized Fatty Acids in Rats. J. Agric. Food Chem..

[9] Penumetcha M., Khan N., Parthasarathy S. (2000). Dietary oxidized fatty acids: An atherogenic risk?. J. Lipid Res..

[10] Staprans I., Rapp J.H., Pan X.M., Kim K.Y., Feingold K.R. (1994). Oxidized lipids in the diet are a source of oxidized lipid in chylomicrons of human serum. Arterioscler. Thromb. Vasc. Biol..

[11] Ng C.-Y., Leong X.-F., Masbah N., Adam S.K., Kamisah Y., Jaarin K. (2014). Heated vegetable oils and cardiovascular disease risk factors. Vascul. Pharmacol..

[12] Khan-Merchant N., Penumetcha M., Meilhac O., Parthasarathy S. (2002). Oxidized fatty acids promote atherosclerosis only in the presence of dietary cholesterol in low-density lipoprotein receptor knockout mice. J. Nutr..

[13] Staprans I., Pan X.M., Rapp J.H., Feingold K.R. (2005). The role of dietary oxidized cholesterol and oxidized fatty acids in the development of atherosclerosis. Mol. Nutr. Food Res..

[14] Chao P.-M., Huang H.-L., Liao C.-H., Huang S.-T., Huang C. (2007). A high oxidised frying oil content diet is less adipogenic, but induces glucose intolerance in rodents. Br. J. Nutr..

[15] Rosen E.D., Sarraf P., Troy A.E., Bradwin G., Moore K., Milstone D.S., Spiegelman B.M., Mortensen R.M. (1999). PPARγ is required for the differentiation of adipose tissue in vivo and in vitro. Mol. Cell.

[16] Tontonoz P., Spiegelman B.M. (2008). Fat and Beyond: The Diverse Biology of PPARγ. Annu. Rev. Biochem..

[17] Varga T., Czimmerer Z., Nagy L. (2011). PPARs are a unique set of fatty acid regulated transcription factors controlling both lipid metabolism and inflammation. Biochim. Biophys. Acta BBA Mol. Basis Dis..

[18] Marion-Letellier R., Savoye G., Ghosh S. (2016). Fatty acids, eicosanoids and PPAR gamma. Eur. J. Pharmacol..

[19] Itoh T., Fairall L., Amin K., Inaba Y., Szanto A. (2008). Structural basis for the activation of PPARγ by oxidized fatty acids. Nat. Struct. Mol. Biol..

[20] Hara A., Radin N.S. (1978). Lipid extraction of tissues with a low-toxicity solvent. Anal. Biochem..

[21] Washburn K.W. (1989). A Modification of the Folch Method of Lipid Extraction for Poultry. Poult. Sci..

[22] Woo K.L., Kim M.C., Ki J.I. (2002). Study on Hydrolysis Method for Extremely Small Amount of Lipids by Organic Basic Sloution, Tetramethylammonium Hydroxide/Methanol and Capillary Gas Chromatographic Analysis of Fatty Acid Composition Depending on Derivatization Methods. J. Appl. Sci..

[23] Ebrahimzadeh A., Pirzad F., Tahanian H., Aghdam M.S. (2019). Influence of Gum Arabic Enriched with GABA Coating on Oxidative Damage of Walnut Kernels. Food Technol. Biotechnol..

[24] Mehyar G.F., Al-Ismail K., Han J.H., Chee G.W. (2012). Characterization of Edible Coatings Consisting of Pea Starch, Whey Protein Isolate, and Carnauba Wax and their Effects on Oil Rancidity and Sensory Properties of Walnuts and Pine Nuts. J. Food Sci..

[25] Nichols P., Dogan L., Sinclair A. (2016). Australian and New Zealand Fish Oil Products in 2016 Meet Label Omega-3 Claims and Are Not Oxidized. Nutrients.

[26] Jairoun A.A., Shahwan M., Zyoud S.H. (2020). Fish oil supplements, oxidative status, and compliance behaviour: Regulatory challenges and opportunities. PLoS ONE.

[27] Imran M., Anjum F.M., Ahmad N., Khan M.K., Mushtaq Z., Nadeem M., Hussain S. (2015). Impact of extrusion processing conditions on lipid peroxidation and storage stability of full-fat flaxseed meal. Lipids Health Dis..

[28] Wang W., Li Y., Cai L., Fang L. (2020). Characteristics on the oxidation stability of infant formula powder with different ingredients during storage. Food Sci. Nutr..

[29] Jackson V., Penumetcha M. (2019). Dietary oxidised lipids, health consequences and novel food technologies that thwart food lipid oxidation: An update. Int. J. Food Sci. Technol..

[30] Jasani B., Simmer K., Patole S.K., Rao S.C. (2017). Long chain polyunsaturated fatty acid supplementation in infants born at term. Cochrane Database Syst. Rev..

[31] Dordevic D., Kushkevych I., Jancikova S., Zeljkovic S.C., Zdarsky M., Hodulova L. (2020). Modeling the effect of heat treatment on fatty acid composition in home-made olive oil preparations. Open Life Sci..

[32] Koelmel J.P., Aristizabal-Henao J.J., Ni Z., Fedorova M., Kato S., Otoki Y., Nakagawa K., Lin E.Z., Godri Pollitt K.J., Vasiliou V. (2021). A Novel Technique for Redox Lipidomics Using Mass Spectrometry: Application on Vegetable Oils Used to Fry Potatoes. J. Am. Soc. Mass Spectrom..

[33] Ciavarella C., Motta I., Valente S., Pasquinelli G. (2020). Pharmacological (or Synthetic) and Nutritional Agonists of PPAR-γ as Candidates for Cytokine Storm Modulation in COVID-19 Disease. Molecules.

[34] Bansal G., Zhou W., Barlow P., Joshi P.S. (2010). Review of rapid tests available for measuring the quality changes in frying oils and comparison with standard methods. Crit. Rev. Food Sci. Nutr..

[35] Ribeiro Filho H.V., Bernardi Videira N., Bridi A.V., Tittanegro T.H., Helena Batista F.A., de Carvalho Pereira J.G., de Oliveira P.S.L., Bajgelman M.C., Le Maire A., Figueira A.C.M. (2018). Screening for PPAR Non-Agonist Ligands Followed by Characterization of a Hit, AM-879, with Additional No-Adipogenic and cdk5-Mediated Phosphorylation Inhibition Properties. Front. Endocrinol..

